# Percutaneous transhepatic management of biliary strictures in patients with dysfunctioning plastic biliary endoprostheses

**DOI:** 10.55730/1300-0144.5430

**Published:** 2022-04-02

**Authors:** Umut ÖĞÜŞLÜ, Gürkan DANIŞAN, Burçak GÜMÜŞ

**Affiliations:** 1 Department of Radiology, Faculty of Medicine, Okan University, İstanbul, Turkey; 2 Department of Radiology, Faculty of Medicine, Sakarya University, Sakarya, Turkey

**Keywords:** Plastic biliary endoprostheses, malignant biliary stricture, benign biliary stricture, percutaneous transhepatic biliary drainage

## Abstract

**Background/aim:**

To evaluate the safety and outcomes of percutaneous transhepatic management of dysfunctioning plastic biliary endoprostheses (PBE) in patients with benign/malign biliary strictures.

**Materials and methods:**

Twenty-nine patients (22 men, 7 women; mean age of 60.7 (range 33–88) years) diagnosed with dysfunctioning PBE were included. Percutaneous transhepatic biliary drainage and subsequent PBE dislodgment into the bowel were performed in all cases. Patient demographics, etiology of the biliary stricture, indication, technical success, complications, and clinical outcomes were gleaned from medical records.

**Results:**

Seventeen patients had malignant strictures, while 12 patients had benign conditions. A total of 36 PBE (33 straight, 3 double-J) were treated. Six patients had more than one PBE. Successful dislodgement of the PBE was achieved in 28 (96.6%) of the cases. Monorail threading was performed in 8 cases while dislodgement by balloon friction was utilized in 21 patients. There was no statistical significance between benign and malignant biliary strictures regarding dislodgement duration (p = 0.080). No major complication was encountered. Thirteen minor complications in 10 patients including abdominal pain (n = 8) and mild hemobilia (n = 5) were observed and treated conservatively. Uneventful passage of the PBE was reported by all patients with technical success.

**Conclusion:**

Percutaneous transhepatic methods aid as a reasonable alternative in the treatment of benign and malignant biliary strictures in patients with dysfunctioning PBE when endoscopic approaches fail or are not eligible.

## 1. Introduction

Biliary strictures occur as a consequence of either benign or malign conditions. Endoscopic plastic biliary endoprostheses (PBE) placement is a minimally invasive procedure and is accepted as the first-line treatment in the management of biliary obstructions [1–3]. Stent dysfunction may be encountered due to migration, impaction, or more commonly occlusion [4]. Proximal migration may result with impaction of the PBE into the biliary tree and/or even erode the adjacent structures which may cause pseudoaneurysms, bleeding, biloma, and abscess formation. Also, dysfunctioning PBE play a role as a nidus for infection [5].

Dysfunctioning PBE are successfully removed and exchanged in the majority of the cases (80%–90%) by endoscopic methods with low complication rates (0%–2%) [6]. However, endoscopic removal of the proximally migrated and impacted PBE might be challenging especially in postoperative patients with distorted anatomy [7]. Percutaneous transhepatic route was pronounced useful as the next step before open surgical removal [8]. However, published data is still limited, and previous reports mostly focused on the management of dysfunctioning PBE in malignant strictures [9–13].

The aim of this study was to present our experience in the percutaneous management of either benign or malign biliary strictures in patients with dysfunctioning PBE.

## 2. Materials and methods

### 2.1. Study design and patient population

The study was designed as a retrospective file review and approved by the ethics committee. Between December 2017 and May 2021 percutaneous transhepatic treatment was performed in 29 consecutive patients who presented with dysfunctioning PBE. Twenty-two (75.9%) of the patients were male. The mean age was 60.7 (range 33–88) years. Seventeen (58.6%) patients had malignant biliary stricture and 12 patients had benign conditions. The indication of PBE placement is detailed in Table 1.

Jaundice was the most frequent symptom and was observed in 27 patients. Other symptoms included fever (n = 14), pain (n = 11), pruritus (n = 7). Recurrent cholangitis was the indication in 4 patients while one patient presented with biliary sepsis.

A total of 36 PBE were treated. More than one PBE had been placed in 6 (20.6%) of the patients including 5 patients with benign biliary strictures and one with hilar cholangiocarcinoma. Thirty-three (83.3%) of the PBE were straight type while three PBE were double-J type. None of the patients had a history of metallic stenting. Obstruction level was distal common bile duct (n = 9), mid-level (n = 1), proximal common bile duct (n = 3), hilum (n = 9) and hepaticojejunostomy anastomosis site (n = 4). Multilevel obstruction was observed in three patients. The mean duration between PBE placement and percutaneous intervention was 45.3 (range 12–100) days. Ultrasound examination was performed on all patients. In selected cases, contrast-enhanced computed tomography (CT) or magnetic resonance imaging of the abdomen was also scheduled. Informed consent was obtained from each patient prior to intervention.

### 2.2. Percutaneous transhepatic technique

All the procedure was performed as outpatient care in a multistep fashion. Routine blood tests, liver function tests, and coagulation parameters were obtained in each step. First, percutaneous biliary drainage was performed. A peripheral branch of the biliary tree was punctured with a 21G needle under sonographic guidance. Guidewire (0.018″) was inserted and the needle was exchanged with a coaxial device (AccuStick II introducer system; Boston Scientific, Marlborough, USA) to upsize the system. Guidewire (0.035″) was inserted and the outer sheath was replaced with 5F angled tip diagnostic catheter (Kumpe; Cook Medical, Bjaeverskov, Denmark) to pass into the bowel, adjacent to the endoprostheses. 8–10F 35cm internal-external drainage catheters (Flexima; Boston Scientific, Marlborough, MA, USA) were placed at operators’ discretion.

The second session of the procedure was performed one week later. 8F sheath was placed and cholangiography was performed to confirm biliary decompression. Subsequently, an attempt was made to pass the 0.035″ guidewire (Radifocus; Terumo, Tokyo, Japan) through the side hole of the endoprostheses and exiting from the end hole as a monorail system. 5F catheters were used to push the endoprostheses into the bowel. In patients with multiple PBE or whom monorail threading technique failed, the guidewire was advanced into the bowel adjacent to the endoprostheses and exchanged with a stiff guidewire (Amplatz Super Stiff guidewire; Boston Scientific, Marlborough, USA). Subsequently, 7–10mm diameter with 4–8 cm length balloon catheters (Sterling and/or Mustang; Boston Scientific, Marlborough, MA, USA) were inflated adjacent to the prostheses to dislodge by friction down into the bowel. A completion cholangiogram was obtained and 10–14F internal-external drainage catheters were placed.

**Figure 1:**
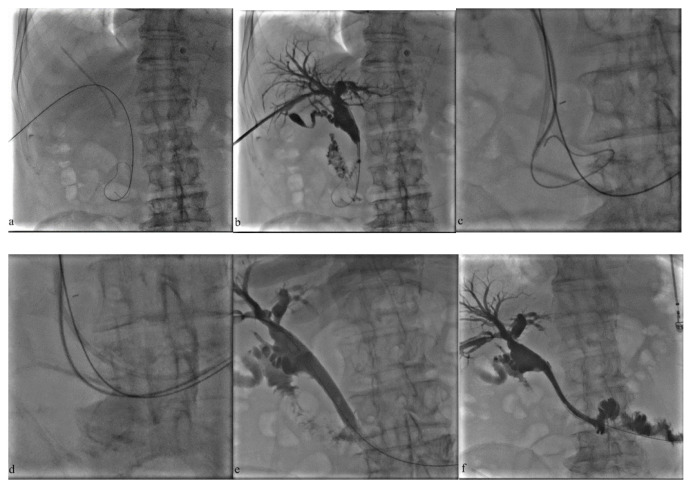
A 84-year-old male with a history of pancreatic adenocarcinoma was admitted with jaundice and pain. **a, b**. The right bile duct was catheterized and cholangiogram revealed proximal migration and impaction of the plastic biliary endoprostheses (PBE) **c, d**. Guidewire was passed through the distal side hole of the PBE and monorail threading with a 5F diagnostic catheter was performed to dislodge the PBE into the duodenum. **e, f**. Endobiliary RFA and metallic stenting were adjusted. Completion cholangiogram shows free contrast media passage.

At the following sessions, stenotic segments were ablated with endobiliary radiofrequency ablation (RFA) probe (ELRA, Starmed, South Korea) and 8–10 mm self-expandable nitinol stents (Protégé™ GPS, Medtronic, Plymouth, Minnesota, USA; Innova™, Boston Scientific, Marlborough, USA) were placed in patients with malignant biliary strictures (Figures 1a–1f).

**Figure 2:**
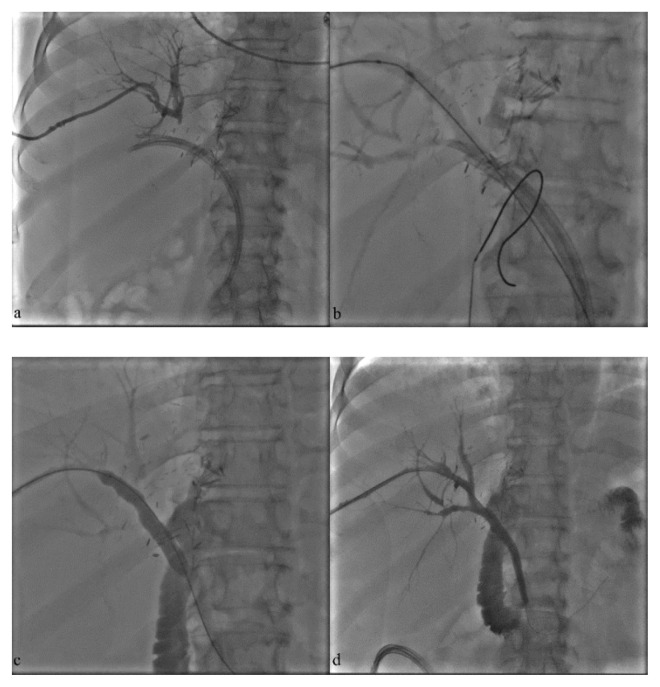
A 67-year-old man with a history of liver transplantation presented with jaundice, fever, pruritus. **a**. Cholangiogram after catheterization of the right bile duct depicts occlusion at the hilar level and distal placement of the plastic biliary endoprostheses (PBE) in the common bile duct. **b**. Dislodgement by friction was performed subsequent to balloon inflation just proximal to the PBE. **c, d**. Balloon angioplasty was performed with 10–12 mm balloons in multiple sessions and completion cholangiogram shows resolution of the stenosis and free passage of the contrast media.

In patients with benign strictures, multiple balloon angioplasty sessions were scheduled to maintain adequate bile flow. In cases with refractory stenosis, an adjunctive endobiliary RFA session was planned in addition to balloon angioplasty (Figures 2a–2d).

### 2.3. Definitions and outcomes

A high-level stricture was defined as the level above the conjunction of the cystic duct to the common hepatic duct. Technical success was defined as successful placement of the percutaneous transhepatic biliary drainage catheter and dislodgement of the PBE into the bowel. Clinical success was defined as relief of the symptoms and normalization of the liver function tests.

Complications were classified according to SIR guidelines [14].

### 2.4. Follow-up

Follow-up was scheduled at the first week, 1, 3, 6 months, and annually thereafter. Signs of either bowel obstruction or perforation were noted during PBE passage. Routine blood tests, liver function tests were obtained and ultrasound examination was performed in each visit. Patients with signs of recurrent obstruction or cholangitis were scheduled for further intervention.

### 2.5. Statistical analysis

Categorical variables are presented as numbers (percentages), and continuous variables are summarized as means (range). The chi-square test was employed for comparisons of categorical variables; nonparametric tests (the Mann-Whitney U test) were used for comparisons of continuous variables. A p value <0.05 was considered statistically significant.

## 3. Results

Endoscopic removal of the prosthesis failed in 21 patients. The remaining patients were referred to the interventional radiology department as the clinicians’ discretion.

Patient demographics, lesion characteristics, and procedural details are summarized in Table 2. Biliary drainage was performed through the right lobe of the liver in 11, left-sided approach was utilized in 14 patients. Bilateral drainage catheters were placed in 4 patients. Intrahepatic bile ducts were dilated in 25 cases while biliary drainage was performed through nondilated bile ducts in 4 cases. All patients with nondilated bile ducts had benign biliary stricture who had been treated with more than one PBE placement.

Proximally migrated PBE were observed in 4 cases. Ineffective drainage due to distal migration was observed in 5. Successful dislodgment of the prosthesis into the bowel was achieved in 28 (96.6%) patients. First attempt was successful in 27 cases, while second attempt was required in one patient with advanced pancreatic carcinoma with double-J PBE. Monorail threading technique was adjusted in 8 (27.6%) cases. Dislodgement by balloon friction was implemented in case of failure of the passage of the guidewire through the side holes of the straight PBE due to severe instent obstruction (n = 13), presence of multiple PBE (n = 6), and double-J PBE (n = 3). In one patient, the distal part of the PBE was impacted in the third part of the duodenum and dislodgement maneuvers failed. The patient was referred to the surgery department and open surgical exploration proved perforation of the duodenum by the PBE. Extraction and primary repair were performed.

The mean procedural duration was 18.1 (range 5–40) min. There was no statistical significance between benign and malignant biliary strictures regarding dislodgement duration (p = 0.080). No major complication was encountered. Minor complications including moderate to severe abdominal pain (n = 8) and mild hemobila (n = 5) were observed in 10 patients and treated conservatively. Hemobilia resolved in the postoperative first week in all patients.

Among the malignant biliary stricture group, further endobiliary RFA and self-expandable metallic stent placement were performed in 6/17 (35.3%) and 13/17 (76.5%) of the cases, respectively. Bilateral stenting was adjusted in four cases.

Among the benign biliary stricture group, common bile duct stones were also displaced into the duodenum in the subsequent session in one patient (no: 9) with a history of cholecystectomy. Repetitive balloon angioplasty sessions were performed in all. Endobiliary RFA was performed in addition to balloon angioplasty due to recalcitrant stenosis in one case (no: 20) with a history of liver transplant.

Mean follow-up was 22.8 (range 2–63) months. None of the patients showed bowel obstruction during PBE passage. Late complication was encountered in 7 patients. Instent occlusion was observed in 3/16 (18.8%) of the patients in the malignant group. While 4/12 (33.3%) of the patients showed signs of recurrent stenosis in the benign stricture group. Repeat drainage was performed in all. During follow-up pertaining to nonprocedural-related causes, 11/28 (39.2%) of the patients died.

## 4. Discussion

Our study demonstrated that treatment of the dysfunctioning PBE through the percutaneous transhepatic route is a safe, effective procedure in both malignant and benign biliary strictures. A high technical success rate of 28/29 (96.6%) was achieved. No major complication was observed even in cases with nondilated bile duct for whom multiple passes were required.

Endoscopic PBE insertion is accepted as the first-line palliative treatment in the management of jaundice in both benign and malignant biliary strictures [3]. Though placement of one stent might be efficient in malignant conditions, multiple stent insertion is the recommended treatment in benign strictures [15]. However, complications of the PBE are not rare. Occlusion was reported at a rate of 18%–23%, and proximal or distal migration was reported between 4.9% and 5.9%, respectively [4,16,17]. Proximal migration may result in impaction which makes endoscopic methods inconvenient [6]. Endoscopic removal and exchange are successful in up to 90% of the cases even in certain difficulties [18]. Duodenal obstruction due to benign or malign conditions such as periampullary diverticulum, surgically distorted anatomy or advanced tumor involvement of the periampullar region may preclude endoscopic approaches [3].

Percutaneous methods have some distinct advantages and drawbacks. Multiple treatment methods can be adjusted over the same access which decreases operation duration in further steps. In the malignant biliary strictures, endobiliary RFA followed by metallic stenting enables a larger lumen up to 10 mm which is greater than PBE [9]. Repetitive balloon angioplasty sessions with or without adjunctive endobiliary RFA, or absorbable stent placement can be performed in benign conditions [15,19]. One major drawback of percutaneous catheter placement is the need for additional care and the uncomfortable nature of the patient. In cases with a nondilated bile duct, multiple punctures might be required which may increase the rate of complications. In the present study, 11/17 (64.7%) patients in the malignant group underwent metallic stenting and repetitive balloon angioplasty was performed in all patients with benign biliary strictures. Adjunctive endobiliary RFA was performed on 6 and one patients in the malignant and benign group, respectively.

Several methods have been described in the percutaneous management of dysfunctioning PBE [8]. Both dislodgements into the bowel and pull-out through hepatic tract techniques were adjusted [11,12,20]. Brown et al. used over-the-wire technique in 36 PBE in 34 patients. Thirty-three patients had malignant biliary obstruction. They achieved to push down the PBE into the bowel except for one case in which PBE could not be dislodged distally even in two sessions [12]. In a series of 43 patients with malignant biliary obstruction, Gümüş reported successful removal of PBE through transhepatic tract in 6 cases without major complication [9]. In the current study, no major complication was observed which is consistent with previous reports. Minor complications were observed in ten cases however managed conservatively.

Type and number of the PBE affect the choice of the percutaneous treatment strategy. Either coaxial or monorail threading methods might be used primarily in straight PBE with flanges which will reduce the cost. Performing catheterization maneuvers to advance the guidewire through side hole of the PBE are easier in the common bile duct than in the bowel due to its narrower lumen. However, these techniques are quite difficult to apply in double-J EBPs and patients with multiple PBE. Dislodgement by friction or transhepatic retrieval could be a better option in these scenarios [8]. In the current study, 33/36 (91.7%) of the PBE were straight and the coaxial/monorail threading technique was successfully applied in 8 cases (malignant = 7, benign = 1) with straight PBE. Since 5/12 (41.6%) of the benign stricture group had multiple PBE, balloon friction was preferred first to reduce the operation duration.

Each method has its attendant drawbacks. PBE are relatively soft products and passes through the gastrointestinal tract uneventfully. Thus, endoscopic removal is not required in the majority of the cases after dislodgement sessions. However, instent sludge formation may harden the stent that may incite bowel perforation during the intestinal passage, or cause bowel obstruction [21,22]. In this study, endoscopic removal and dislodgement techniques failed in one case with malignant biliary stricture which had been treated with straight PBE placement. Surgical exploration revealed duodenal perforation. Performing maneuvers with a snare in the biliary tree may be traumatic when removal through the transhepatic tract is considered [8]. Larger sheaths and a mature tract are required in pulling out techniques to avoid potential complications including bleeding, pseudoaneurysm formation, and bile leak. However, when the distal or proximal end of the PBE is not eligible to be snared, off-label use of biopsy or bronchial forceps were described to grab and pull out the PBE which may impregnate potential complications, prolong operation duration, increase radiation dose and cost [10,23].

There are limitations in this study. The number of patients is limited to generalize our results. Straight type PBE had been placed in the majority of the patients so comparing the treatment of different types of PBE was not possible. Dislodgement into the bowel was the chosen strategy in all patients. Adding pulling out through transhepatic tract techniques might influence outcomes. However, in contrast with previous reports benign conditions were also included and treated successfully.

In conclusion, though endoscopic methods are successful to remove dysfunctioning PBE in the majority of the cases, percutaneous transhepatic methods emerge as a reasonable alternative in the treatment of biliary strictures when endoscopic approaches fail or are not eligible.

## Figures and Tables

**Table 1:** Indications for plastic biliary endoprostheses placement.

Indication		Number (%)
Benign		12 (41.4)
	Living-donor-transplantation	5 (17.2)
	Left-hepatectomy	3 (10.4)
	Cholecystectomy	4 (13.8)
Malign		17 (58.6)
	Pancreatic adenocarcinoma	8 (27.6)
	Cholangiocarcinoma	8 (27.6)
	Gastric-antrum adenocarcinoma	1 (3.4)

**Table 2:** Patient demographics and procedural details.

Variable		Benign	Malignant	Total	*P* value
Number of patients		12 (41.4)	17 (58.6)	29 (100)	-
Sex					0.927
	Male	9 (75)	13 (76.5)	22 (75.9)	
	Female	3 (25)	4 (23.5)	7 (24.1)	
Age (years)					0.027
	Mean ± SD	54.3 ± 3.0	65.3 ± 2.9	60.7	
	Range	33–67	45–88	33–88	
Number of PBE					-
	1	7 (58.3)	16 (94.1)	23 (79.3)	
	2	4 (33.3)	1 (5.9)	5 (17.2)	
	3	1 (8.3)	0	1 (3.4)	
Type of PBE					-
	Straight	16 (88.9)	17 (94.5)	33 (83.3)	
	Double-J	2 (11.1)	1 (5.5)	3 (16.7)	
Interval between PBE insertion and removal (days)					0.811
	Mean ± SD	46 ± 7.2	44.9 ± 6.0	45.3 ± 24.5	
	Range	15–93	12–100	12–100	
Technical success		12 (100)	16 (94.1)	28 (96.6)	
Treatment method					0.051
	Monorail threading	1 (8.3)	7 (41.2)	8 (27.6)	
	Balloon friction	11 (91.7)	10 (58.8)	21 (72.4)	
Operation duration (min)					0.080
	Mean ± SD	21.3 ± 2.3	15.9 ± 2.5	18.1 ± 9.6	
	Range	15–40	5–36	5–40	
Complication					-
	Major	0	0	0	
	Minor	4 (100)	6 (100)	10 (100)	

Note–Values in parentheses indicates percentages. SD = Standard deviation, PBE = Plastic billiary endoprosthesis.
